# Will you still need me, will you still feed me when I'm 64? The health impact of caregiving to one's spouse

**DOI:** 10.1002/hec.3542

**Published:** 2017-09-21

**Authors:** P.L. de Zwart, P. Bakx, E.K.A. van Doorslaer

**Affiliations:** ^1^ University Medical Center Groningen Groningen The Netherlands; ^2^ Institute of Health Policy and Management Erasmus University Rotterdam (EUR) Rotterdam The Netherlands; ^3^ Erasmus School of Economics EUR Rotterdam The Netherlands; ^4^ Tinbergen Institute Rotterdam The Netherlands

**Keywords:** informal care, long‐term care, propensity score matching, SHARE

## Abstract

Informal care may substitute for formal long‐term care that is often publicly funded or subsidized. The costs of informal caregiving are borne by the caregiver and may consist of worse health outcomes and, if the caregiver has not retired, worse labor market outcomes. We estimate the impact of providing informal care to one's partner on the caregiver's health using data from the Survey of Health, Ageing, and Retirement in Europe. We use statistical matching to deal with selection bias and endogeneity. We find that in the short run caregiving has a substantial effect on the health of caregivers and, for female caregivers, on their health care use. These effects should be taken into account when comparing the costs and benefits of formal and informal care provision. The health effects may, however, be short‐lived, as we do not find any evidence that they persist after 4 or 7 years.

## 1 INTRODUCTION

Informal caregiving to frail elderly by family or friends is often regarded as an important way to sustainably meet the rising demand for long‐term care, in particular because its direct costs appear much lower than the costs of formal care provided by professionals: Informal caregivers generally do not receive monetary compensation (OECD, 2011). Even though informal care is usually unpaid, its provision may entail substantial direct and indirect opportunity costs, both for the caregiver and for the health care budget: It may negatively affect the mental and physical health of the caregiver, and these health problems may, combined with time constraints, in turn lead to increased health spending, absenteeism, or even reduced labor force participation.

We study the impact of providing informal care on the health and health care use of caregiving spouses using data from the Survey on Health, Ageing and Retirement in Europe (SHARE). Spouses provide about a third of all informal care (OECD, 2011) while they are likely to have retired and to be in frail health themselves. Hence, for this subgroup, the caregiving tasks may be more burdensome and the health effects more severe than for younger caregivers. Appropriate justification of long‐term care policy requires a thorough understanding of all relevant costs and benefits associated with the provision of formal and informal care. The health effects of caregiving may be substantial and difficult to compensate monetarily, and therefore, we need evidence on the significance and relevance of these health effects. Moreover, if caregivers seek treatment for any health problems caused by their caregiving status, high rates of caregiving may also be bad news for the public purse, as the greater share of health care is publicly funded in many European countries.

### 1.1 The decision to provide informal care

In a simple version of a Roy model (Borjas, 1987; Heckman & Honoré, 1990; Heckman & Sedlacek, 1985), individuals choose between a paid job and home production, which includes caregiving tasks. 1We assume that the amount of informal care provided by other family members is exogenous and insufficient. The additional payoff of having a paid job is *D*, which is a random variable reflecting differences in ability. But a paid job also involves a cost *C* of hiring a formal caregiver, which is influenced by the government through subsidization 2The governments of virtually all European countries pay for at least some formal long‐term care (OECD, 2011). but is a given (i.e., exogenous) to individuals. As a result, the utility maximizing individual favors home production as long as *D* < *C* and favors taking up a paid job otherwise. As ability and health status are correlated, we need to account for selection when studying the effect of caregiving on health.

If the government subsidizes formal care more (thus lowering *C*), we expect the share of caregivers in the population and the number of hours provided by these caregivers to be lower. Hence, if the health effects *H* of an additional hour of caregiving are individual‐specific but do not depend on the total number of hours of caregiving, we expect that the average health effect of being a caregiver is larger when (a) *C* is higher and (b) when the demand for informal care is higher, that is, when the health problems of the spouse are more severe.

In addition to differences in *C* and *D*, the decision between formal and informal care may also be affected by factors influencing the nonmonetary cost of the two alternatives. The latter group of factors may include the expected health costs of providing informal care and cultural differences—including gender patterns—changing the perception of potential caregivers of what is expected from them. 3Additionally, gender patterns in caregiving may partially persist when there is a gender pay gap on the labor market. These cultural differences may thus lead to differences in the propensity to provide care and the amount of care provided and thus to differences between countries and between men and women.

Most individuals providing care to their spouse are retired. However, the retired face a related choice: to enjoy leisure time and hire a formal caregiver or to give up leisure time to provide informal care to their spouse. Hence, with diminishing marginal benefits from leisure and increasing marginal disutility of informal care provision and in the presence of individual‐level differences in (a) the marginal decrease in utility resulting from providing care (e.g., because of functional limitations and bad health 4Note that health, which is correlated with ability, may have opposite effects before and after retirement.) and (b) the value of an additional unit of leisure (e.g., because of differences in income and wealth), the decision to provide informal care will be affected by these characteristics. As these characteristics also influence the health of the caregiver, this self‐selection into informal caregiving needs to be accounted for when studying the effect of caregiving on health, as in the simplified Roy model.

### 1.2 Previous findings

Three recent studies have addressed the endogeneity caused by selection bias using an instrumental variable approach to estimate a causal effect between caregiving and caregiver's health (Coe & Van Houtven, 2009; Do, Norton, Stearns, & Van Houtven, 2015; Heger, 2016). 5A related strand of the literature, which deals with a similar endogeneity issue, studies the impact of caregiving on labor market outcomes. Recent contributions include Crespo and Mira (2014), Skira (2015), and Van Houtven, Coe, and Skira (2013). Using data from the US Health and Retirement Survey on the characteristics of siblings and the death of the mother as instrumental variables, Coe and Van Houtven (2009) find significant negative effects on mental health and self‐reported health (married respondents) and heart conditions (single men), both immediately and a few years later (depressive symptoms in married women only). Specifically, they find a 15% increase in the mean score of their depression measure due to the onset of caregiving. Using South Korean data, Do et al. (2015) instrument daughter‐in‐law's informal caregiving status by their parents‐in‐law's health endowments and conclude that caregiving increases the probability of reporting pain affecting daily activities, a fair or poor self‐rated health and the out‐of‐pocket spending on outpatient care by the caregiver when seeking treatment. Heger (2016) studies the impact of providing care to a parent on being depressed for European SHARE respondents aged 50 to 70 years using fixed effects and using changes in the number of parents alive as an instrument. Her fixed‐effects instrumental variable analysis shows a large effect on depressive symptoms for female caregivers but no effect for males.

Part of the differences in the findings may be the result of differences in the specific subpopulations studied—Coe and Van Houtven (2009) include all adults, Do et al. (2015) only include married women, Heger (2016) only adults aged 50 to 70 years—and the fact that they use different instruments and thus estimate local average treatment effects for different groups of “compliers”: respondents whose caregiving status is influenced by the instrument. The instruments used in these studies only apply to the case in which the children provide care to parents. Children are an important source of caregiving but still make up only about a third of all caregivers (OECD, 2011).

Several other studies have addressed the endogeneity of caregiving using fixed effects regressions (Kaschowitz & Brandt, 2017; Leigh, 2010; Schmitz & Stroka, 2013; Van den Berg, Fiebig, & Hall, 2014). These fixed‐effects regressions yield mixed results: Although Leigh (2010) finds no effect on life satisfaction, Van den Berg et al. (2014) report a negative impact. Kaschowitz and Brandt (2017) find negative effects on the mental health and self‐reported health for caregiving within the household using data from England and continental Europe and mixed results for caregiving to someone in another household.

Finally, some studies have used propensity score matching. 6Lechner (2009) provides a discussion of the advantages of statistical matching over fixed‐effects regressions.
^,^ 7There is quite some variety in the study populations of these studies (and the fixed‐effects regression studies). Leigh (2010) and Van den Berg et al. (2014) use Australian data, whereas Schmitz and Stroka (2013), Stroka (2014), and Schmitz and Westphal (2015) use German data. Brenna and Di Novi (2016), Heger (2016), and Kaschowitz and Brandt (2017) use data from multiple European countries. Schmitz and Stroka (2013) find an effect on the use of antidepressants and tranquilizers for those who provide care while working full time but not on drugs for physical health problems, suggesting that caregiving affects mental health only. Stroka (2014) uses the same data, but a broader study population, and reconfirms the increase in antidepressants and tranquilizers, but also finds an effect on painkillers and gastrointestinal agents, which suggests an increase in physical health problems. Schmitz and Westphal (2015) arrive at the same conclusion using the mental and physical scales underlying the SF‐12 composite health measure that is collected as part of the German Socio‐Economic Panel; Brenna and Di Novi (2016) analyze one cross‐section of SHARE data and confirm that caregivers are more likely to show depressive symptoms, but only find a significant effect for caregivers in southern European countries. Specifically, they find an increase in depression of about 24% when compared to the mean southern European sample.

We contribute to the existing literature in two respects. First, we study the effects on health of providing informal caregiving to a spouse, in contrast to all of the above‐mentioned studies, which either do not specify the relationship between caregiver and care recipient or focus on caregiving of children to parents or parents‐in‐law. 8Brenna and Di Novi (2016) study caregivers providing care to a parent; Van den Berg et al. (2014) include caregivers to parents, parents‐in‐law, spouses, and other disabled adult relatives, but do not separate them. Leigh (2010), Schmitz and Stroka (2013), Stroka (2014), Schmitz and Westphal (2015), and Kaschowitz and Brandt (2017) cannot or do not specify the care recipient and thus calculate an effect averaged over all types of care recipients. Spouses make up about a third of the total group of caregivers (OECD, 2011). The effects on health and health care use may be larger for those providing care to a spouse than for those providing care to a parent for three reasons (Pinquart & Sörensen, 2011): Because spouses are most likely to live with the care recipient, they tend to provide more hours of caregiving and find less respite from their caregiver role than other caregivers. Moreover, their closer relationship to the care recipient might in itself be the cause of additional stress. Finally, a spouse caregiver is generally older and in worse health than a child caregiver to a parent and hence spouse caregiver health may be more frail.

Second, we are the first to describe how attrition affects the estimates of the long‐term effects of caregiving when panel survey data are used—and may have affected results in prior studies. Selective attrition is a common problem when using panel data from a survey, but one that has not received attention in previous studies.

## 2 DATA

### 2.1 Sample

We use waves 1–2 and 4–5 from SHARE. 9This study is based on version 2.6.1 of the first (2004) and second (2006) waves, version 1.1.1 of the fourth (2010) wave and version 1.0.0 of the fifth (2013) wave. See Börsch‐Supan et al. (2013) for methodological details.
^,^ 10Although information on life histories from the retrospective third wave (SHARELIFE) could potentially be valuable, we chose not to add it because of attrition and because the life history information appeared to be only distantly related. SHARE respondents are people aged 50 years and older and their spouses. The first two waves contain 47,100 individuals (68,263 observations) from 11 countries. 11The 11 countries are Austria, Belgium, Switzerland, Germany, Denmark, Spain, France, Greece, Italy, the Netherlands, and Sweden. Israel is excluded because the timing of data collection in Israel deviated from that in other countries. However, we exclude all respondents who (a) are not present in both waves 1 and 2, (b) who live without a spouse (including those living separate from their spouse), (c) for whom some information is missing, (d) or who provided care to their spouse in wave 1. 12The vast majority of the observations are removed for reasons (a) and (b) meaning that the results are likely to be still representative for all spousal caregivers (see Appendix). After removal of these individuals, the final dataset that is used for the matching contains 10,472 individuals, with roughly equal numbers of men (5,185) and women (5,108). Outcome data for waves 4 and 5, collected approximately 4 and 7 years after wave 2, are used to estimate the longer term effects of caregiving. Because of attrition, the sample size falls in wave 4 (to 5,992) and wave 5 (to 5,229).

### 2.2 Model and variables

Respondents are classified as a caregiver when they provided any help to their partner daily or almost daily during at least 3 months within the past 12 months, where help is defined as personal care, for example, washing, getting out of bed, or dressing.

We study the impact of caregiving on four outcome measures: (a) whether the respondent uses prescription drugs, (b) the number of doctor visits in the past 12 months, (c) depression as measured by the EURO‐D scale (Prince et al., 1999) ranging from 0 (*not depressed at all*) to 12 (*severely depressed*), 13The Euro‐D scale is a 12‐item questionnaire specifically designed to measure depression late in life. All 12 items have weight 1, meaning the score ranges from 0 to 12 (Prince et al., 1999). and (d) self‐perceived health (SPH) on a 5‐point scale. 14SPH is expected to pick up both health and well‐being effects. Caregiving may affect the well‐being of a spousal caregiver in two ways. That is, in addition to the direct effect of caring for one's spouse, there is a “family effect”: a caregiver may care about the care recipient (Bobinac, van Exel, Rutten, & Brouwer, 2010). In this article, however, the latter effect is partly mitigated by matching on the partner's health status at wave 1.


To ensure high‐quality matching, it is important to include information about all characteristics affecting both one's health and whether one is a caregiver. From Section 1, it follows that there are a number of factors affecting the decision to provide informal care. Schmitz and Westphal (2015) summarize these as follows: (a) the need to provide care, (b) the willingness to provide care, and (c) the ability to provide care.

The need to provide care to the partner is related to characteristics of the partner, as well as to the availability of alternative sources of care (both formal and informal). Hence, we include information on the age and the health of the partner. The availability of formal care is proxied by indicators for the region of Europe in which the respondent lives, 15The 11 included countries are clustered in a northern, central and southern European region, using the same clusters as Brenna and Di Novi (2016) based on long‐term care spending and historical differences in the welfare state. The northern region includes Denmark, Sweden and the Netherlands, the central region consists of Austria, Belgium, France, Germany and Switzerland, while the southern region comprises Italy, Spain, and Greece.
^,^ 16Using (a) propensity scores estimated using country dummies and (b) exact matches on country dummies yields poorer matches but final results that are not much different from the preferred estimates (available upon request). by household income and wealth (which may also proxy for willingness to provide care) and the availability of informal care by the presence of children living at home, the total number of children, the share of these children who are daughters, and the number of siblings.

How respondents react to a demand for care (i.e., by providing informal care or hiring a professional caregiver) depends on (a) their ability to provide informal care and (b) their willingness to give up leisure when retired or paid labor otherwise. The respondent's ability may be proxied by age; gender; whether he provided informal care at wave 1 and his health status; and the willingness to provide informal care by employment status, education level, income, wealth, and proxies for personality.

## 3 METHODS

The decision to provide informal care depends, among other things, on the ability of the potential caregiver to provide care and on his or her opportunity cost of time (Section 1). To address any selection bias, we use statistical matching. That is, every individual providing informal care is matched to a set of individuals not providing informal care with similar observable characteristics. These two matched groups together then form a reduced sample in which being a caregiver is uncorrelated with the other observed characteristics (and therefore the propensity to become a caregiver is equalized). To estimate the average treatment effect of caregiving on health in this sample, we perform a regression analysis to control for any residual differences between the treatment group and the controls (Stuart, 2010).

### 3.1 Identifying assumptions

Matching yields an unbiased estimate of the treatment effect if two assumptions hold (Rosenbaum & Rubin, 1983). The first assumption is the Stable Unit Treatment Value Assumption, which requires that there is a unique health outcome *h*_*iT*_ for individual *i* and caregiving activity *T* and that this outcome does not depend on treatment assignment (i.e., caregiving activity) of another individual *j*. In our case, the health outcome of one partner is certainly dependent on the caregiving activity of the other partner. Therefore, this assumption only holds if partners are separated during the analysis, which is achieved by sample stratification with respect to gender. 17This strategy does not separate same‐sex couples. However, their number is very small (40 households in 2004) and thus has a negligible impact on the results.
^,^ 18This separation is also useful because it yields results that are easily comparable to the existing literature, as virtually all other studies study health effects either on men and women separately (e.g., Coe and Van Houtven (2009)) or on women only (e.g., Brenna and Di Novi (2016); Do et al. (2015); Schmitz and Westphal (2015)).


The second assumption is that of a Strongly Ignorable Treatment Assignment (SITA). This assumption has two components. First, no combination of covariates should be fully predictive of caregiving or noncaregiving to one's partner. For this reason, individuals living without a partner are removed from the dataset before matching.

Second, there should be no relevant remaining unobserved differences between the matched groups of caregivers and noncaregivers, conditional on the observed covariates. To fully satisfy this assumption, all information related to both health and caregiving should be included. Typically, information on some characteristics is not available, yet this may not be a problem because these unobserved characteristics are often correlated with observed characteristics (Stuart, 2010). Therefore, matching on observed covariates implies at least some degree of matching on unobserved covariates that are correlated with the observed ones, and hence, this assumption is reasonable when the set of observed characteristics is fairly complete.

To further increase the likelihood that this assumption holds, we exploit the fact that respondents are interviewed four times and follow the same approach as Lechner (2009) and Schmitz and Westphal (2015): We use the first wave to define the covariates and the second wave to define the treatment status. We then match respondents providing care in the second wave to respondents not providing care in the second wave. The two main advantages of this strategy are (a) that the treatment status cannot influence the covariates and (b) that we can control for differences in prior health status and health care use by including lagged dependent variables to estimate the propensity score. Consequently, time‐invariant heterogeneity is highly unlikely to play a role: Such heterogeneity would already have affected the health status and health care use in the first wave.

Another advantage of this strategy is that we may stratify by previous caregiving activity. This is likely to capture much of the unobserved (possibly health‐related) heterogeneity affecting caregiving activity in later years (and thus treatment assignment as well). Moreover, this stratification thus mitigates endogeneity caused by persistence in caregiving (Lechner, 2009). Therefore, the SITA assumption is much more likely to hold if matched individuals have the same previous caregiving status. We focus on the stratum not providing care in wave 1 in the remainder of this article, because the stratum providing care in wave 1 is too small to perform a similar analysis.

The second wave is also used to assess the immediate impact of caregiving, whereas the last two waves are only used to estimate the longer‐term impact. 19Respondents in the treatment group and in the control group may or may not be caregivers in subsequent waves. This means that the analysis presented here thus resembles an intention to treat analysis as the health status in the later waves may be affected by differences in caregiving activities in subsequent waves. The results presented here are nonetheless meaningful because individuals face a choice to become a caregiver at the second wave without being able to foresee how long they will be providing care. To maximize the sample size in each of the analyses, the matching is performed separately to determine the immediate, medium‐term (4 years), and long‐term effects (7 years).

### 3.2 Matching procedure

Including more information improves the quality of the matching and makes it more likely for the SITA assumption to hold. However, it also complicates the matching: Exact matching on all variables is not feasible when the number of variables on which respondents are matched is large. Instead, we match on the propensity score, that is, the probability of being a caregiver conditional on the relevant covariates, which is estimated using a probit regression. Propensity scores have two key properties. First, matching on the propensity score ensures that the conditional distribution of the observed covariates given the propensity score is the same for caregivers as for noncaregivers. Second, if the SITA assumption holds given the covariate vector *X*, then it also holds given the propensity score (Rosenbaum & Rubin, 1985). In sum, the propensity score summarizes all relevant covariate information in a single value while not compromising on the necessary assumptions (Stuart, 2010).

We match observations using kernel weighting matching: Each treated observation *i* is matched to multiple nontreated individuals, with weights determined by the absolute difference in propensity scores with individual *i* and the particular kernel function that is used. The main advantage of this method is that few individuals need to be excluded from the analysis, and thus, little information is lost. A disadvantage is that it requires that nontrivial decisions are made regarding the matching process. 20Choices have to be made about the value of a bandwidth parameter and the type of kernel function. The bandwidth measures how similar the propensity scores of two individuals should be for them to be regarded as a match. The choice of bandwidth quantifies the trade‐off between bias and variance of treatment effect estimates. The bandwidth is set at 0.03 because this bandwidth is the smallest bandwidth for which many treated individuals can be matched. The kernel used is a standard Epanechnikov kernel.


Following Rubin (2001), we test the similarity of the covariate distributions using three statistics: (a) the absolute standardized difference of the means of the propensity score between the treated and matched control group (Rubin's B), (b) the ratio of variance of the propensity score of the treated and the matched control group (Rubin's R), and (c) the ratio of the variances of the residuals from regressions of each of covariates on the propensity score for the treated and the control group. 21The samples are usually sufficiently balanced when the first statistic is smaller than 0.25 and the second statistic is between 0.5 and 2 (Rubin, 2001). The third statistic is between 0.8 and 1.25 for variables for which the matching was successful.


To calculate the average treatment effect on the treated, 22The analysis is restricted to the treated individuals for whom suitable matches were found, and hence, the ATT is only applicable to these subgroups. Table 3 reports the number of matched and unmatched individuals. the outcome is regressed on the treatment indicator and all covariates for the matched sample using the weights from the kernel matching as probability weights. Through this regression, we correct for small residual variation in covariate distributions between the matched groups when determining the average treatment effect on the treated (Lechner, 2009; Rubin, 1973). All these regressions are estimated with cluster‐robust standard errors at the household level.

As described above, roughly 40% of the respondents who were interviewed in wave 1 and wave 2 were not interviewed in wave 4. Hence, the estimates of the longer term effects of caregiving may be influenced by selective attrition. If the effect of caregiving on health is not of equal size for all subgroups of respondents and if some of the subgroups for which the effect is expected to be particularly large are more likely to drop out, attrition may lead to an underestimation of the effect of caregiving on health. To examine whether selective attrition is a threat to the identification of long‐term effects, we analyze whether there is an association between the propensity to be a caregiver and the probability of dropping out of the sample after wave 2.

## 4 RESULTS

### 4.1 Descriptive statistics and the propensity to provide care

Compared to noncaregivers, caregivers in wave 2 are older, have an older spouse, and are in worse health in wave 1 (Table 1). Furthermore, caregivers earn lower incomes and are less likely to be highly educated than noncaregivers. These differences highlight that selection bias may affect the results and therefore needs to be addressed.

**Table 1:** Descriptive statistics

Variable	Total	Noncaregivers	Caregivers
*Treatment indicator*			
Caregiver to spouse in wave 2	0.040	0	1***
*Health outcomes in wave 2*			
Depressive symptoms	1.948	1.904	3.025***
% Using prescription drugs	0.701	0.693	0.872***
Number of doctor visits	5.769	5.642	8.808***
Self‐perceived health	2.035	2.061	1.428***
*Health outcomes in wave 4*			
Depressive symptoms	2.227	2.199	2.919***
% Using prescription drugs	0.783	0.779	0.893***
Number of doctor visits	6.344	6.254	8.576***
Self‐perceived health	1.950	1.966	1.551***
*Health outcomes in wave 5*			
Depressive symptoms	2.266	2.233	3.141***
% Using prescription drugs	0.798	0.794	0.906***
Number of doctor visits	6.575	6.522	7.955***
Self‐perceived health	2.066	2.050	2.487***
*Covariates (measured in wave 1)*			
Depressive symptoms	1.990	1.965	2.591***
Having ≥1 activity limitation	0.636	0.644	0.452***
Doctor visits	5.268	5.170	7.589***
Prescription drug use	0.661	0.654	0.823***
Self‐perceived health	2.203	2.224	1.704***
Age	62.049	61.799	67.998***
Number of children	2.316	2.319	2.260
Living with a child	0.308	0.314	0.168***
Number of daughters	1.181	1.182	1.147
Number of siblings	4.838	4.846	4.648
Education: low	0.479	0.474	0.608***
Education: medium	0.315	0.317	0.270**
Education: high	0.205	0.209	0.123***
Fraction of household income earned by respondent	0.201	0.206	0.095***
Log of standardized household income	9.602	9.612	9.361***
Assets (in 10.000 PPP‐adjusted euros)	36.386	36.716	28.542**
Employed	0.332	0.341	0.106***
Retired	0.454	0.445	0.669***
Homemaker	0.160	0.161	0.151
Unemployed	0.054	0.053	0.073
Motivation for charity or voluntary work: contribute to something useful	0.219	0.220	0.199
Motivation for charity or voluntary work: I am needed	0.284	0.287	0.217***
Caregiver to a parent	0.089	0.089	0.069
Living in northern Europe	0.282	0.285	0.213***
Living in central Europe	0.443	0.442	0.473
Living in southern Europe	0.275	0.274	0.314*
Living in an urban environment	0.481	0.483	0.426**
Age of the spouse	61.967	61.683	68.716***
Depressive symptoms of the spouse	1.934	1.897	2.811***
Spouse activity limitations	0.651	0.642	0.884***
Spouse prescription drug use	5.116	4.971	8.570***
Spouse doctor visits	0.645	0.659	0.319***
Spouse self‐perceived health	2.208	2.239	1.478***
Number of observations[Link]	10,471	10,048	423

* differences with the sample of noncaregivers are significant at *p* < 0.10.

** differences with the sample of noncaregivers are significant at *p* < .05.

*** differences with the sample of noncaregivers are significant at *p* < .01.

Number of observations is smaller for health outcomes in wave 4 and wave 5 (see main text).

In the study sample, 4.3% of the women and 3.6% of the men are caregivers at *t* = 0. The probit regressions of caregiver status on covariates, which are used to estimate the propensity score, 23As explained in Section 3, the propensity score is estimated separately for each of the samples. To save space, Table 2 contains the estimates for the sample used to estimate the immediate impact of caregiving on health for females who did not provide care in wave 1 only; the estimates for the other samples, which are very similar, are available upon request. show (in all six study samples) that having a spouse in bad health and of high age is strongly positively associated with being a caregiver, as is the case for being retired or unemployed (Table 2). For the study sample used to estimate the short‐term effect of caregiving for women presented in Table 2, the propensity to be a caregiver is furthermore positively associated with a higher probability of prescription drug use before the onset of the caregiving spell and a higher fraction of total household income earned by the respondent, whereas living together with a child is associated with a lower propensity to provide care to one's spouse. 24There are minor differences between the six study samples (full results available upon request); the covariates listed here are not associated with being a caregiver for all study samples. Conversely, other covariates are associated with caregiving in the other study sample that are not listed here, including age, family composition indicators, and being a caregiver to a parent or parent‐in‐law.


**Table 2:** Propensity score estimation

	Coefficient
Depressive symptoms	0.004
Having ≥1 activity limitation	−0.101
Doctor visits	0.002
Prescription drug use	0.165*
Self‐perceived health	−0.014
Age	−0.035
Age^2^	0.000
Number of children	−0.031
Living with a child	−0.191*
Number of daughters	−0.039
Number of siblings	0.013
Education: medium	0.017
Education: high	−0.049
Fraction of household income earned by respondent	0.640**
Log of standardized household income	−0.051
Assets (in 10.000 PPP‐adjusted euros)	0.000
Retired	0.439***
Homemaker	0.260
Unemployed	0.540***
Motivation for charity or voluntary work: contribute to something useful	0.072
Motivation for charity or voluntary work: I am needed	−0.129
Caregiver to a parent	0.142
Living in northern Europe: Denmark, Sweden, or the Netherlands	−0.079
Living in southern Europe: Greece, Italy, or Spain	0.002
Living in an urban environment	−0.071
Age of the spouse	0.021***
Depressive symptoms of the spouse	0.036*
Spouse prescription drug use	0.323***
Spouse doctor visits	0.008
Spouse activity limitations	−0.246***
Spouse self‐perceived health	−0.140***
Intercept	−1.779
Number of observations	5,108

*Notes*: Results from the propensity score estimation for the subgroup of male respondents for the estimates of the immediate effects. The estimates of the propensity scores for the other subgroups are available upon request.

* differences with the sample of noncaregivers are significant at *p* < .10.

** differences with the sample of noncaregivers are significant at *p* < .05.

*** differences with the sample of noncaregivers are significant at *p* < .01

Some of the treated individuals are removed because their estimated propensity score is too high to be reliably matched to untreated individuals. However, the share of individuals that is removed is less than 1% (Table 3).

**Table 3:** Matched sample

			Treated observations	
		Total Observations	Matched	Not matched
Males	Immediate	5,184	185	0
	4 years	2,858	95	3
	7 years	2,499	76	1
Females	Immediate	5,108	220	0
	4 years	3,025	133	0
	7 years	2,767	114	0

The matching procedure (full details available upon request) mitigates virtually all differences in the means of observed characteristics between the treated and control group: The absolute standardized differences in the means decrease to zero for the propensity score and covariates. Finally, the ratio of the variance of regression residuals between the treated and control group indicates that many covariates were classified as “of concern” or “bad” before the matching on the basis of this ratio, whereas only very few are still of concern after the matching. For instance, in the case of the analysis of the immediate impact on female caregivers, the variance ratio for 18 covariates is “of concern” or “bad” before, and only the variance of standardized household income remains a concern after matching. On the basis of these results (available upon request), we conclude that the matching sufficiently reduces the differences between the treatment and control groups.

### 4.2 Short‐term effects on health and health care use

The regressions on the matched sample show a substantial and significant immediate effect of caregiving on the health of caregivers (Table 4). For females, it leads to more symptoms of depression; for this subgroup, caregiving causes an immediate increase of 0.570 points of the depression score on a scale of 0 to 12. The full sample mean is 1.948, implying an increase of about 29%. Considering that the difference between a person with no symptoms of depression and a person that is likely to be clinically identified as depressed is only 4 points (Prince et al., 1999) on this scale, providing care to the partner may have a substantial contribution. Our estimate is larger than the estimates reported by Brenna and Di Novi (2016) and Coe and Van Houtven (2009) who find increases in their depression measures of 24% (in southern Europe only) and 15%, respectively, but smaller than the 38% increase reported by Heger (2016). Furthermore, caregiving is also found to lead to worse self‐reported health. For males, the effects are smaller but significant, and Heger (2016) does not find any impact of caregiving on depressive symptoms for men. In addition to negative health effects, we find that caregiving leads to a higher probability of using prescription drugs (6.2 percentage points increase) and 1.4 more doctor visits per 12 months for female caregivers (about a 26% increase from the mean of 5.3), and Do et al. (2015) do not find effects on use of these types of health care. 25The other articles studying the impact on medicine use—Schmitz and Stroka (2013) and Stroka (2014)—do find positive effects, but their outcome measures are incomparable with our estimates. This increase in health care use can be a direct result of the lowered health, although it is also possible that the confrontation with health problems while caring for an ill person leads to larger care use in itself. Thus, our outcomes suggest that at least for some dimensions—health effects for males and health care use for females—care provision to a spouse is indeed more burdensome than caregiving to others, and for female caregivers, the magnitude of the health effects of caregiving to one's spouse is similar to those found for other types of caregiving.

**Table 4:** Estimation results

		Depressive symptoms	Self‐reported health	Prescription drug use	Doctor visits	*n*
Males	Immediate	0.453*** (0.161)	−0.161** (0.069)	0.002 (0.023)	0.666 (0.497)	5,181
	4 years	−0.184 (0.180)	0.068 (0.089)	−0.018 (0.032)	0.877 (0.641)	2,953
	7 years	0.150 (0.232)	0.020 (0.098)	−0.015 (0.036)	1.216 (0.787)	2,575
Females	Immediate	0.570*** (0.157)	−0.202*** (0.061)	0.062*** (0.019)	1.365*** (0.474)	5,107
	4 years	−0.095 (0.179)	0.014 (0.073)	0.011 (0.024)	0.014 (0.517)	3,024
	7 years	−0.133 (0.199)	0.017 (0.075)	0.043** (0.020)	−1.537*** (0.580)	2,764

*Notes*: The covariates used for these regressions were the same as those used for the propensity score estimation, shown in Table 2, measured during the first wave. Standard errors in parentheses.

* the results are significant at *p* < .10.

** the results are significant at *p* < .05.

*** the results are significant at *p* < 0.01.

### 4.3 Longer‐term effects and selective attrition

We find no health effects at all after 4 and 7 years. There are some effects on health care use for females: Prescription drug use is higher and the number of doctor visits is lower after 7 years.

The absence of health effects in the long run is not due to a lack of power as the estimates are almost as precisely estimated as the immediate effects. Rather, it appears to be the result of selective attrition: Individuals with higher propensity scores are indeed somewhat more likely to drop out after wave 2 (Table 5). Ill health and use of care of the partner are the most important determinants of the higher propensity scores (Table 2) and disproportionate dropout of individuals with partners with greater care burden lowers the average care burden in the later waves. If care burden is associated with negative health effects (as it is hypothesized to be), it is likely that the negative intermediate‐term and long‐term effects of providing care to the partner on the caregiver's health are underestimated in this study, especially for men.

**Table 5:** Sensitivity test for selective attrition

		Full sample	Respondents present in waves 4 and 5
Females	Mean propensity in 2006	0.043 ± 0.053	0.040 ± 0.055
	Effect of caregiving on depressive symptoms	0.570*** (0.157)	0.557** (0.232)
	Effect of caregiving on self‐reported health	−0.202*** (0.061)	−0.134* (0.08)
	Effect of caregiving on prescription drug use	0.062*** (0.019)	0.050* (0.026)
	Effect of caregiving on doctor visits	1.365*** (0.474)	0.304 (0.640)
	Number of observations	5,107	2,463
Males	Mean propensity in 2006	0.036 ± 0.044	0.028 ± 0.035
	Effect of caregiving on depressive symptoms	0.453*** (0.161)	0.000 (0.188)
	Effect of caregiving on self‐reported health	−0.161** (0.069)	−0.153 (0.109)
	Effect of caregiving on prescription drug use	0.002 (0.023)	−0.013 (0.035)
	Effect of caregiving on doctor visits	0.666 (0.497)	0.066 (0.687)
	Number of observations	5,181	2,291

*Notes*: The column “Full sample” contains the estimates of immediate effects from Table 4. Standard errors are in parentheses.

* the results are significant at *p* < .10.

** the results are significant at *p* < .05.

*** the results are significant at *p* < .01.

A comparison of the original estimates of the immediate effect with estimates of the immediate effect of caregiving on health for the subsample that is also interviewed in wave 4 and wave 5 shows that this is indeed the case: The point estimates of the immediate health effects of caregiving in Table 5 for the subsample (column 2) are always smaller than the estimates for the full sample (column 1) that, together with larger standard errors caused by a smaller sample size of the subsample interviewed in all four waves that we use, leads to fewer statistically significant results. So although the findings for the full sample suggest that the health effects of caregiving disappear over time, it may be that selective attrition has played a role in this phenomenon. 26The comparison between the mean propensity for the full sample and for the subsample that is present in waves 4 and 5 shows that the attrition rate is higher in the group that has a relatively high propensity to care. This finding suggests that if respondents with a higher propensity to care face more serious health consequences of caregiving tasks, for example, because their spouse has more severe functional limitations, their higher attrition rate may be causing the lack of the long‐run effects. An additional logit regression of attrition on all observed characteristics (available upon request) shows that at a more detailed level, attrition is associated with being male, being in better health, age, not cohabiting with children, having more siblings, fewer years of education, earning a larger fraction of total household earnings, being retired, being a homemaker and being unemployed (compared to being employed), doing voluntary work because one wants to contribute to society, living in northern or southern Europe (compared to living in central Europe), having a younger partner, and having a partner who visits a doctor less frequently.


## 5 CONCLUSION AND DISCUSSION

Informal caregivers provide much of the help with daily activities that frail elderly receive, and the great majority of this care is provided by family members. Caregiving is time‐consuming and may be burdensome, and any such negative effects need to be accounted for when comparing the costs and benefits of formal and informal care. The impact of caregiving on the caregiver is also relevant as both economic theory and empirical evidence suggest that because of self‐selection into caregiving any adverse health effects of caregiving will make socioeconomic gradients in health and income steeper. The effects of caregiving by spouses on their health and health care use are of particular interest because spouses are likely to have retired and to be older and in relatively worse health when they start to provide care. Hence, this subgroup of caregivers is likely to experience more severe effects of caregiving on their health and health care use. To estimate the effects of caregiving by spouses on their health and health care use, we exploit the availability of exceptionally good information on the health, well‐being, and activities of both spouses in the SHARE data. We use this information to overcome endogeneity bias through statistical matching.

The main findings are as follows. First, for women and men alike, caregiving leads to an increase in depressive symptoms and to a reduction in self‐perceived health. These effects are larger for women than for men; for example, women face an increase in depressive symptoms of about 0.57, and the score for men increases by 0.45, which is large relative to the full sample mean of 1.948. Second, for women, caregiving significantly raises their use of medical care: On average, it increases the probability for a woman to use prescription drugs by 6.2 percentage points and leads to 1.4 more doctor visits per year. As expected, the effects on health and health care use are at least similar but often larger than the effects estimated in studies that included (or solely focused on) caregivers who helped neighbors, friends, and other family members than their spouses. Estimates of these effects on health and health care use facilitate a more informed comparison of the costs and benefits of formal and informal care provided by spouses, which is relevant input for policy decisions that influence the mix of informal and formal care through subsidies and other incentives (OECD, 2011). Effects on health and health care use may play a particularly important role in such a cost–benefit analysis because they may worsen existing inequalities in health and because health effects are more difficult to compensate than reduced labor market opportunities, as monetary transfers are less likely to suffice as compensation for health losses than for a reduction in labor supply.

Third, we find that the short‐term negative effects on health and health care use do, however, disappear over time: 4 and 7 years after the treatment assignment, the health and health care use of the respondents who were caregivers initially were not significantly different from those of the matched control group. The finding that the negative health effects disappear over time is in line with the findings of Schmitz and Westphal (2015) for German caregivers (spouses and others). There may be three reasons for this. First, it may be that the caregiving activities are temporary and that their effects do not last. Second, caregivers may find ways to cope with the burden of caregiving. Third, selective attrition may have biased the results: The immediate effects of caregiving are smaller and insignificant for the subsample that is interviewed four times. Selective attrition is a major issue with any panel survey data and may thus also have affected the results from previous studies that aim to identify long‐run effects of caregiving using similar data.

Next to attrition, the data we use have two other limitations. First, despite the extensive background information that we used, there may be unobserved characteristics or circumstances that may cause violations of the SITA on which unbiasedness of the results is dependent. This assumption is inherently untestable, but in this case, the main threats may be a lack of detailed information on the quality of life beyond self‐reported health and a lack of more direct measures of personality traits affecting both the propensity to caregiving and the health and health care use. Second, measures of both care provision and health are self‐reported and crude and therefore measurement error and aggregation may have biased the results.

As the identification of longer term effects will bring us a step closer to a balanced weighting of all costs associated with informal care provision, future research should focus on potential solutions to avoid the problem of selective attrition in panel surveys. In this context, the growing availability of administrative population register data with information about long‐run health outcomes is one possible avenue to explore.

## 1

**Table A1:** Descriptive statistics sample selection

	Number of observations
Wave 2 sample	37,447 (100%)
Interviewed in wave 1 too	21,163 (56.5%)
Living with a spouse	13,798 (36.8%)
No information is missing	10,847 (29.0%)
Did not provide care to spouse in wave 1	10,472 (28.0%)

% indicates the percentage of the original wave 2 sample that is left after applying the sample selection criterion

## References

[hec3542-bib-0001] Bobinac, A. , van Exel, N. , Rutten, F. , & Brouwer, W. (2010). Caring for and caring about: Disentangling the caregiver effect and the family effect. Journal of Health Economics, 29(4), 549–556. 20579755 10.1016/j.jhealeco.2010.05.003

[hec3542-bib-0002] Borjas, G. (1987). Self‐selection and the earnings of immigrants. American Economic Review, 77, 531–553.

[hec3542-bib-0003] Börsch‐Supan, A. , Brandt, M. , Hunkler, C. , Kneip, T. , Korbmacher, J. , Malter, F. , … Zuber, S. (2013). Data resource profile: The Survey of Health, Ageing and Retirement in Europe (SHARE). International Journal of Epidemiology, 42(4), 992–1001. 23778574 10.1093/ije/dyt088 PMC3780997

[hec3542-bib-0004] Brenna, E. , & Di Novi, C. (2016). Is caring for older parents detrimental to women's mental health? The role of the European North‐South gradient. Review of Economics of the Household, 14(4), 745–778.

[hec3542-bib-0005] Coe, N. , & Van Houtven, C. (2009). Caring for mom and neglecting yourself? The health effects of caring for an elderly parent. Health Economics, 18(9), 991–1010. 19582755 10.1002/hec.1512

[hec3542-bib-0006] Crespo, L. , & Mira, P. (2014). Caregiving to elderly parents and employment status of European mature women. Review of Economics and Statistics, 96(4), 693–709.

[hec3542-bib-0007] Do, Y. K. , Norton, E. , Stearns, S. , & Van Houtven, C. (2015). Informal care and caregiver's health. Health Economics, 224–237. 24753386 10.1002/hec.3012 PMC4201633

[hec3542-bib-0008] Heckman, J. , & Honoré, B. (1990). The empirical content of the Roy model. Econometrica, 58, 1121–1149.

[hec3542-bib-0009] Heckman, J. , & Sedlacek, G. (1985). Heterogeneity, aggregation, and market wage functions: An empirical model of self‐selection in the labor market. Journal of Political Economy, 93, 1077–1125.

[hec3542-bib-0010] Heger, D. (2016). The mental health of children providing care to their elderly parent. Health Economics, https://doi.org/10.1002/hec.3457. 10.1002/hec.3457 27917556

[hec3542-bib-0011] Kaschowitz, J. , & Brandt, M. (2017). Health effects of informal caregiving across Europe: A longitudinal approach. Social Science and Medicine, 173, 72–80. 27930918 10.1016/j.socscimed.2016.11.036

[hec3542-bib-0012] Lechner, M. (2009). Long‐run labour market and health effects of individual sports activities. Journal of Health Economics, 28(4), 839–854. 19570587 10.1016/j.jhealeco.2009.05.003

[hec3542-bib-0013] Leigh, A. (2010). Informal care and labor market participation. Labour Economics, 17(1), 140–149.

[hec3542-bib-0014] OECD . (2011). Help wanted? Providing and paying for long‐term care. Paris: OECD.

[hec3542-bib-0015] Pinquart, M. , & Sörensen, S. (2011). Spouses, adult children, and children‐in‐law as caregivers of older adults: A meta‐analytic comparison. Psychology and Aging, 26(1), 1–14. 21417538 10.1037/a0021863 PMC4449135

[hec3542-bib-0016] Prince, M. , Reischies, F. , Beekman, A. , Fuhrer, R. , Jonker, C. , Kivela, S. , … Van Oyen, H. (1999). Development of the EURO‐D scale: A European Union initiative to compare symptoms of depression in 14 European centres. The British Journal of Psychiatry, 174(4), 330–338. 10533552 10.1192/bjp.174.4.330

[hec3542-bib-0017] Rosenbaum, P. , & Rubin, D. (1983). The central role of the propensity score in observational studies for causal effects. Biometrika, 70(1), 41–55.

[hec3542-bib-0018] Rosenbaum, P. , & Rubin, D. (1985). Constructing a control group using multivariate matched sampling methods that incorporate the propensity score. The American Statistician, 39(1), 33–38.

[hec3542-bib-0019] Rubin, D. (1973). The use of matched sampling and regression adjustment to remove bias in observational studies. Biometrics, 29(1) 185–203.

[hec3542-bib-0020] Rubin, D. (2001). Using propensity scores to help design observational studies: Application to the tobacco litigation. Health Services and Outcomes Research Methodology, 2(3–4), 169–188.

[hec3542-bib-0021] Schmitz, H. , & Stroka, M. (2013). Health and the double burden of full‐time work and informal care provision: Evidence from administrative data. Labour Economics, 24, 305–322.

[hec3542-bib-0022] Schmitz, H. , & Westphal, M. (2015). Short‐ and medium‐term effects of informal care provision on female caregiver's health. Journal of Health Economics, 42, 174–185. 25968112 10.1016/j.jhealeco.2015.03.002

[hec3542-bib-0023] Skira, M. (2015). Dynamic wage and employment effects of elder parent care. International Economic Review, 56(1), 63–93.

[hec3542-bib-0024] Stroka, M. (2014). The mental and physical burden of caregiving—Evidence from administrative data. Ruhr economic papers 474.

[hec3542-bib-0025] Stuart, E. (2010). Matching methods for causal inference: A review and a look forward. Statistical Science, 25(1), 1. 20871802 10.1214/09-STS313 PMC2943670

[hec3542-bib-0026] Van den Berg, B. , Fiebig, D. , & Hall, J. (2014). Well‐being losses due to care‐giving. Journal of Health Economics, 35, 123–131. 24662888 10.1016/j.jhealeco.2014.01.008 PMC4051992

[hec3542-bib-0027] Van Houtven, C. , Coe, N. , & Skira, M. (2013). The effect of informal care on work and wages. Journal of Health Economics, 32(1), 240–252. 23220459 10.1016/j.jhealeco.2012.10.006

